# Fever in Africa

**Published:** 1846-06

**Authors:** 


*Fever in Africa.*—The days of out-and-out Broussaism are, thank God, passed; and medical men, even in France, have now found out that a fever is not necessarily an inflammation. The possession of Algieria, if it has not been very useful to our neighbors in a commercial or political point of view, has at least, had the effect of teaching the medical officers of their army—and the important lesson has gradually extended itself to the civil practitioners—to abandon many of the principles of their early professional education in Paris, and to have recourse to a more enlightened and successful method of treating the fevers of Africa, which are almost invariably of a remittent or intermittent character. Bark, opium, and wine, have, in a great measure, taken the place of venesection and ptisans. In these fevers, it is of the highest consequence to keep up the spirits of the patient; for the state of the mind has no inconsiderable influence in aiding or in counteracting the effects of the remedies employed for their subjugation. Often has the expected fit of ague been observed not to occur, if the attention has been intensely occupied by something of absorbing interest, or if the feelings have been strongly roused by some joyous or alarming intelligence.—*Med. Chir. Rev.*

